# Incidence and types of laryngotracheal sequelae of prolonged invasive ventilation in COVID-19 patients

**DOI:** 10.1007/s00405-022-07467-8

**Published:** 2022-06-04

**Authors:** Giacomo Fiacchini, Joel Reuben Abel, Domenico Tricò, Alessandro Ribechini, Rachele Canelli, Miriana Picariello, Fabio Guarracino, Francesco Forfori, Iacopo Dallan, Stefano Berrettini, Luca Bruschini

**Affiliations:** 1 Otolaryngology, Audiology and Phoniatric Operative UnitDepartment of Surgical, Medical and Molecular Pathology and Critical Care MedicineAzienda Ospedaliero-Universitaria Pisana (AOUP), University of Pisa, Via Paradisa, 2, 56124 Pisa, Italy; 2 Department of Surgical, Medical, Molecular Pathology and Critical Area, University of Pisa, Pisa, Italy; 3 Thoracic Endoscopic Unit, Azienda Ospedaliero-Universitaria Pisana, Pisa, Italy; 4 Cardiothoracic and Vascular Anaesthesia and Intensive Care, Department of Anaesthesia and Critical Care Medicine, Azienda Ospedaliero-Universitaria Pisana (AOUP), Pisa, Italy

**Keywords:** COVID-19, SARS-CoV-2, Laryngotracheal lesions, Intensive care unit, Tracheal stenosis

## Abstract

**Purpose:**

The COVID-19 outbreak has led to an increasing number of acute laryngotracheal complications in patients subjected to prolonged mechanical ventilation, but their incidence in the short and mid-term after ICU discharge is still unknown. The main objective of this study is to evaluate the incidence of these complications in a COVID-19 group of patients and to compare these aspects with non-COVID-19 matched controls.

**Methods:**

In this cohort study, we retrospectively selected patients from November 1 to December 31, 2020, according to specific inclusion and exclusion criteria. The follow-up visits were planned after 6 months from discharge. All patients were subjected to an endoscopic evaluation and completed two questionnaires (VHI-10 score and MDADI score).

**Results:**

Thirteen men and three women were enrolled in the COVID-19 group while nine men and seven women were included in the control group. The median age was 60 [56–66] years in the COVID-19 group and 64 [58–69] years in the control group. All the patients of the control group showed no laryngotracheal lesions, while five COVID-19 patients had different types of lesions, two located in the vocal folds and three in the trachea. No difference was identified between the two groups regarding the VHI-10 score, while the control group showed a significantly worse MDADI score.

**Conclusions:**

COVID-19 patients subjected to prolonged invasive ventilation are more likely to develop a laryngotracheal complication in the short and medium term. A rigorous clinical follow-up to allow early identification and management of these complications should be set up after discharge.

## Introduction

The new Coronavirus Disease 2019 (COVID-19), caused by the SARS-CoV-2 virus, requires admission to an intensive care unit (ICU) for massive interstitial pneumonia for up to 12% [1–3] of all patients who contract the infection, with possible orotracheal intubation and subsequent tracheostomy to allow adequate invasive mechanical ventilation. The most common airway-related complications of such ICU maneuvers are laryngotracheal granulomas, scar webs, stenosis, tracheomalacia and, less commonly, tracheal necrosis with tracheo-esophageal or tracheo-arterial fistulas.

According to the current literature, patients subjected to prolonged intubation for COVID-19 interstitial pneumonia have a higher incidence of tracheal complications during hospitalization or in the early post-treatment period than a control population [4–7]. These patients may also have a higher incidence of long-term laryngotracheal sequelae [8]. In fact, it is already well known in the literature that patients undergoing prolonged intubation (> 8 days) have a higher risk of developing both acute and late laryngotracheal complications [9, 10]. For these reasons, the main objective of this study is to evaluate the incidence and the different patterns of laryngotracheal sequelae in a COVID-19 group of patients subjected to prolonged invasive ventilation and to compare these aspects with non-COVID-19 matched controls. Secondly, we administered two specific questionnaires to assess dysphonia and dysphagia in both groups.

## Methods

Subjects were selected retrospectively through our electronic medical records according to the following inclusion criteria: patients with COVID-19 pneumonia (COVID-19 group) or other pathologies (control group) subjected to invasive mechanical ventilation for at least 8 days with orotracheal intubation and/or tracheostomy; patients admitted to our ICUs during the Italian second wave from November 1 to December 31, 2020 for the COVID-19 group and in the same time period for the control group; ≥ 18 years old; ≥ 6 months from the interruption of invasive ventilation; subjects’ consent to study enrolment.

Eligible subjects were invited to come to our outpatient clinic and, after a brief anamnesis, their basic data, Adult Comorbidity Evaluation-27 (ACE-27) [11], days of intubation (orotracheal ± tracheostomy), any laryngotracheal complications arising during hospitalization and any laryngotracheal issues at the time of the clinical evaluation were collected and analyzed. Then, patients completed the Italian version of the Voice Handicap Index-10 (VHI-10) [12]—and the MD Anderson Dysphagia Inventory (MDADI) [13] questionnaires. After that, and after the application of a local anesthetic spray, patients were subjected to a fiberoptic endoscopic evaluation of all the laryngeal subsites and the trachea to identify any possible lesions of these structures.

This study was approved by the Local Ethics Committee on June 24, 2021 (Prot. N° 20147). Written informed consent to collect deidentified data was obtained from all patients. This study followed the Strengthening the Reporting of Observational Studies in Epidemiology (STROBE) reporting guideline.

### Statistical analysis

Continuous variables are reported as median [interquartile range] and categorical variables are reported as count (percentage). Group differences in continuous or categorical variables were tested using Mann–Whitney *U* test or Fisher’s exact test, respectively. Group differences in ordinal variables were also tested using the Cochran–Armitage test for trends. Analyses were performed using JMP Pro 16.0 (SAS, Cary, NC) at a two-sided α level of 0.05.

## Results

We identified 62 consecutive patients with COVID-19 admitted to our ICU in the aforementioned time frame. Forty-one subjects met our inclusion and exclusion criteria. Twenty-five subjects refused to do the follow-up visit or died during the hospital stay or after discharge. The remaining 16 patients completed the clinical evaluation and answered the questionnaires. The same evaluations were performed by 16 control patients matched for age and sex over the same 2-month period admitted to our non-COVID-19 ICUs for different reasons.

**Fig. 1:**
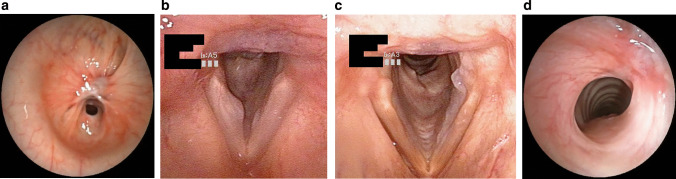
Examples of the laryngotracheal lesions identified. **A** Tracheal stenosis (Myer-Cotton grade III) before tracheal resection. **B** Edematous polyp of the middle and posterior third of the right vocal fold. **C** Left vocal process granuloma and healing ulcer of the middle and posterior third of the left vocal fold. **D** Subglottic stenosis (Myer-Cotton grade I)

Thirteen (81.3%) and nine (56.3%) men were enrolled in the COVID-19 group and the control group, respectively. The median age was 60 [56–66] years in the COVID-19 group and 64 [58–69] years in the control group (Table 1). COVID-19 and control groups were matched for age and sex. Furthermore, no statistical difference was identified between the two groups regarding the ACE-27, the number of tracheostomies performed or the number of days with orotracheal intubation and tracheostomy cannula. Patients in the control group underwent significantly more surgical tracheostomies (eight patients) than in the COVID-19 group (one patient), where ten percutaneous tracheostomies were performed. All COVID-19 patients were subjected to pronation maneuvers, while none was pronated in the control group. Two COVID-19 patients were subjected to surgical procedures after ICU discharge. More precisely, one of them required a tracheal resection with end-to-end anastomosis for severe tracheal stenosis (Myer-Cotton grade III [14]) after the failure of an endoscopic carbon dioxide (CO_2_) laser-assisted procedure (Fig. 1A). The other patient had a symptomatic Myer-Cotton grade II tracheal stenosis which was managed conservatively and successfully with an endoscopic CO_2_ laser-assisted tracheoplasty.

**Table 1:** Comparison of the COVID-19 group with the control group

	COVID-19	Controls	p*	*p* for trend**
Age, years	60 [56–66]	64 [58–69]	0.678	
Men, *n* (%)	13 (81.3)	9 (56.3)	0.252	
ACE-27			0.919	0.574
None	4	4		
Mild	6	4		
Moderate	5	6		
Severe	1	2		
Time after extubation (months)	9.5 [9–10]	9 [9–10]	0.727	
Tracheostomy, *n* (%)	11 (68.8)	15 (93.8)	0.172	
Surgical tracheostomy, *n* (%)	1 (9.1)	8 (53.3)	**0.036**	
Days with orotracheal intubation	8.5 [8–9]	8 [8–9]	0.287	
Days with tracheostomy	13 [11–15]	16.5 [9.8–20.8]	0.287	
Pronation	16 (100)	0 (0)	**< 0.0001**	
Dysphonia	1 (6.3)	0 (0)	0.999	
Dysphagia	2 (12.5)	5 (31.3)	0.394	
VHI-10 Score	0 [0–2]	2 [0–4]	0.317	0.738
MDADI Score	0 [0–1]	2 [0–5]	**0.049**	0.074

Bold = *p* < 0.05

Data are median [interquartile range] or number (percentage)

*Ace-27* Adult Comorbidity Evaluation-27 [11], *VHI-10* Voice Handicap Index-10 [12], *MDADI* MD Anderson Dysphagia Inventory [13]

*Mann–Whitney *U* test or Fisher's exact test

**Cochran Armitage trend test, ordinal numbers

The majority of patients did not complain of dysphonia and dysphagia, without significant differences between the two groups (Table 1).

At the endoscopic evaluation, all the patients showed a normal laryngeal motility and a normal laryngeal space (Table 2). All the patients of the control group showed no laryngotracheal lesions, while 5 (31%) COVID-19 patients had different types of lesions. Specifically, at the level of the glottis, one patient showed an edematous polyp of the middle and posterior third of the right vocal fold (Fig. 1B), while another patient had a left vocal process granuloma and a healing ulcer of the middle and posterior third of the left vocal fold (Fig. 1C). One patient showed a subglottic stenosis (Myer-Cotton grade I) (Fig. 1D), while the other two patients who were submitted to surgical procedures for tracheal stenosis in the period between ICU discharge and our follow-up visit presented the sequelae of these surgeries. The stenoses were located from the first to the third tracheal rings in two patients while the other patient had the subglottic region involved. No patient required medical or surgical interventions after our visits.

**Table 2:** Comparison of the endoscopic findings between the COVID-19 group and the control group

	COVID-19	Controls	p*
Normal laryngeal motility	16 (100)	16 (100)	0.999
Normal laryngeal space	16 (100)	16 (100)	0.999
Total laryngotracheal lesions	5 (31.3)	0 (0)	**0.043**
Supraglottic lesions	0 (0)	0 (0)	0.999
Glottic lesions	2 (12.5)	0 (0)	0.484
Subglottic lesions	1 (6.3)	0 (0)	0.999
Tracheal lesions	2 (12.5)	0 (0)	0.484

Bold = *p* < 0.05

Data are number (percentage)

^*^Mann–Whitney *U* test or Fisher's exact test

No difference was identified between the two groups regarding the VHI-10 score, while the control group showed a significantly worse MDADI score (Table 1).

## Discussion

The COVID-19 outbreak in the pre-vaccine era has led to an increasing number of patients admitted to ICUs who required invasive mechanical ventilation. This has led to re-discussing a series of aspects related to the management of ICU patients, including the exact timing of the tracheostomy after oro-tracheal intubation and the different types of tracheostomy to be performed, specifically percutaneous or surgical.

Prior to the pandemic, Mehel and colleagues[10] concluded that tracheostomy should be performed for intubations that extend for more than 7 days, to minimize the risk of laryngotracheal injuries. On the contrary, with the advent of the SARS-CoV-2 virus, many authors and organizations, such as The American Academy of Otolaryngology-Head and Neck Surgery, recommended that tracheostomy should not be performed prior to 14 days of endotracheal intubation[15] to avoid unnecessary procedures (remember that the median time from hospital admission to death was 5 days in China, six in UK and seven in Italy [15–17]), and to minimize the risk of infection for healthcare professionals by having a reduced viral load.

Regarding the choice between percutaneous or surgical tracheostomy, the whole otorhinolaryngological and anesthesiological community is perfectly aware of the pros and cons of both techniques and issues related to COVID-19 [18–21]. We opted to perform percutaneous tracheostomy with the “Ciaglia Blue Rhino” technique for most COVID-19 patients (91%), a procedure that generates minimal aerosolization; we reserve the surgical technique only for patients with certain characteristics such as difficult neck anatomy, goiter and coagulopathy, following an internal protocol similar to that published by Bassi and colleagues [22]. However, according to the current literature, there is no evidence that one technique generates more laryngotracheal sequelae than the other.

The combination of these aspects led the Laryngotracheal Stenosis Committee of the European Laryngological Society to publish a paper where the authors warned the medical and scientific communities of the possibility of a surge in the number of laryngotracheal sequelae in the short and medium term [8]. The aim of our study was to verify whether an increase in laryngotracheal lesions had actually occurred in COVID-19 patients subjected to mechanical ventilation for at least 8 days compared to a control population.

Even if no statistically significant difference was identified between the two groups with respect to the incidence of the lesions in the various sub-sites of the larynx and trachea, the COVID-19 group showed a statistically significant higher incidence of global laryngotracheal lesions than the control group (5 VS 0, *p* < 0.05), with no difference in terms of number of days with orotracheal intubation, days with tracheostomy and number of tracheostomies performed. However, this did not lead to a higher incidence of dysphagia and dysphonia in the COVID-19 group and did not result in significant differences in the scores on the VHI-10 questionnaire. On the contrary, the MDADI score result was significantly worse in the control group, but we believe this is due to the high scores obtained by patients #2 and #4, admitted to our ICU for ischemic cerebral stroke that left severe neurological sequelae, including dysphagia.

Many etiopathogenetic hypotheses have been proposed to explain the unprecedented increase of acute laryngotracheal complications observed in COVID-19 patients subjected to prolonged invasive mechanical ventilation during their ICU admission [4], and most of them could also explain the onset of laryngotracheal sequelae in short and mid-term after ICU discharge.

In this study, we highlighted how pronation maneuvers could be a determining factor for these types of complications. Specifically, by moving the patient from the supine to the prone position, the orotracheal tube cuff is supposed to increase its pressure on the tracheal walls, thus causing tissue lesions [23]. Other possible etiopathogenetic causes could be the high cuff pressure, the use of large caliber tubes, the microvascular injury of laryngo-tracheal mucosa caused by the prothrombotic and antifibrinolytic state of these patients, the use of high dose systemic steroids, the high viral replication within the laryngotracheal mucosa which could weaken the epithelium itself [24], or unreported mistakes or accidents by physically and emotionally exhausted health care professionals [25]. However, to date, it must be specified that none of these hypotheses has been confirmed.

The current literature [4, 8, 26] proposes a number of recommendations to prevent and manage these types of complications. Specifically, during ICU stay, high steroid dose should be used with caution and the cuff pressure should be monitored periodically, especially after moving the patient from the supine to the prone position or vice versa. Moreover, after ICU discharge, every COVID-19 patient subjected to invasive mechanical ventilation should be followed with periodic flexible fiberoptic nasolaryngoscopy or bronchoscopy to detect any early signs of laryngotracheal lesions and act promptly.

This study has several limitations. The main ones are intrinsic to the retrospective nature of this study and are the lack of data such as the caliber of orotracheal tubes and tracheostomy cannulas and the lack of CT scans for some patients. Another significant limitation is the small sample size. However, it confirms the concerns of the Laryngotracheal Stenosis Committee of the European Laryngological Society about the increased incidence of laryngotracheal complications. Otolaryngologists and Thoracic Surgeons will need to be prepared to manage an increasing number of these complications.

## Conclusions

COVID-19 patients subjected to prolonged (≥ 8 days) invasive mechanical ventilation are more likely to develop a laryngotracheal complication in the short and medium term after discharge than a non-COVID-19 control group. COVID-19 patients should undergo a rigorous clinical follow-up to allow early identification and management of these complications.

## Data Availability

All data generated or analyzed during this study are included in this published article.
